# Detection of acute thoracic aortic dissection based on plain chest radiography and a residual neural network (Resnet)

**DOI:** 10.1038/s41598-022-26486-3

**Published:** 2022-12-19

**Authors:** Dong Keon Lee, Jin Hyuk Kim, Jaehoon Oh, Tae Hyun Kim, Myeong Seong Yoon, Dong Jin Im, Jae Ho Chung, Hayoung Byun

**Affiliations:** 1 Department of Emergency Medicine, Seoul National University Bundang Hospital, Seongnam, Republic of Korea; 2 Department of Emergency Medicine, Seoul National University College of Medicine, Seoul, Republic of Korea; 3 Department of Computer Science, Hanyang University, 222 Wangsimni‑ro, Seongdong‑gu, Seoul, 04763 Republic of Korea; 4 Machine Learning Research Center for Medical Data, Hanyang University, Seoul, Republic of Korea; 5 Department of Emergency Medicine, College of Medicine, Hanyang University, 222 Wangsimni‑ro, Seongdong‑gu, Seoul, 04763 Republic of Korea; 6 Department of Radiology and Research Institute of Radiological Science, Severance Hospital, Yonsei University College of Medicine, Seoul, Republic of Korea; 7 Department of Otolaryngology-Head and Neck Surgery, College of Medicine, Hanyang University, Seoul, Republic of Korea; 8 Department of HY, College of Medicine, KIST Bio-Convergence, Hanyang University, Seoul, Republic of Korea

**Keywords:** Machine learning, Aortic diseases

## Abstract

Acute thoracic aortic dissection is a life-threatening disease, in which blood leaking from the damaged inner layer of the aorta causes dissection between the intimal and adventitial layers. The diagnosis of this disease is challenging. Chest x-rays are usually performed for initial screening or diagnosis, but the diagnostic accuracy of this method is not high. Recently, deep learning has been successfully applied in multiple medical image analysis tasks. In this paper, we attempt to increase the accuracy of diagnosis of acute thoracic aortic dissection based on chest x-rays by applying deep learning techniques. In aggregate, 3,331 images, comprising 716 positive images and 2615 negative images, were collected from 3,331 patients. Residual neural network 18 was used to detect acute thoracic aortic dissection. The diagnostic accuracy of the ResNet18 was observed to be 90.20% with a precision of 75.00%, recall of 94.44%, and F1-score of 83.61%. Further research is required to improve diagnostic accuracy based on aorta segmentation.

## Introduction

Acute thoracic aortic dissection is a life-threatening disease, in which blood leaking from the damaged inner layer of the aorta causes dissection between the intimal and adventitial layers. The diagnosis of this disease is challenging. Although the true incidence rate is difficult to determine, large series of autopsies have reported that the rate of prevalence of aortic dissection is 0.2–0.8%^1^. However, it is one of the most catastrophic cardiovascular diseases, with a mortality rate of 50% within 48 h if not diagnosed and treated properly.

The most common symptoms of aortic dissection are severe chest or back pain of abrupt onset, but they are highly variable between patients^2,3^. A substantial number of patients complain of nonspecific symptoms, such as abdominal pain, nausea, discomfort, and syncope, but some do not report discomfort. In addition, physical examination results are relatively normal. These characteristics make the diagnosis of aortic dissection difficult.

The modality of choice for diagnosing aortic dissection is contrast-enhanced computed tomography (CT)^2^. Contrast-enhanced CT can reliably identify the dissection flap and false lumen, which are the primary diagnostic features of aortic dissection. However, because of possible atypical presentations and other diagnoses that may mimic aortic dissection, diagnostic modalities, such as chest X-ray scanning, laboratory tests including D-dimer, and bed-side transthoracic echocardiography, are used to screen for aortic dissection. Among them, chest X-ray imaging is most commonly used to differentiate the various causes of chest pain rapidly. It is also used as a screening test for acute thoracic aortic dissection. However, the sensitivity of chest X-ray scanning through a widening of the aortic silhouette is only 70%, as reported in previous studies^4,5^.

With recent developments in the application of deep learning technology to image recognition, significant research has been conducted on automatic interpretation of medical images, including X-ray images. In particular, algorithms have been developed to diagnose diseases such as tuberculosis, pneumonia, and pneumothorax using chest X-ray scanning^6–9^.

Given the need for rapid and accurate diagnosis of acute thoracic aortic dissection, the use of deep learning technology may be helpful. During the conception of this study, we hypothesised that the accuracy of diagnosis could be improved by analysing chest x-rays, which are the fastest available screening modality in clinical practice, using deep learning. Therefore, we sought to investigate the accuracy of a deep learning algorithm for screening acute thoracic aortic dissection based on chest X-ray scanning using a Convolutional Neural Network (CNN) model.

### Related works

Contrast CT is the modality of choice used to diagnose aortic dissection. Several studies have been conducted on the detection of thoracic aortic dissection based on CT images using CNN. Recently, Hata et al. investigated the diagnostic performance of a deep learning algorithm for aortic dissection based on CT images. In aggregate, the data of 170 patients were considered, including 85 patients with aortic dissection. Only non-contrast CT images were used, and an area under the curve (AUC) of 0.940 was achieved, alongside an accuracy of 90.0%, sensitivity of 91.8%, and specificity of 88.2%^10^. Another study attempted a similar task based on contrast CT images. The authors constructed a U-Net-based semantic segmentation architecture to segment the aortic lumen and performed aortic circularity analysis on the segmentation results. Their detection results exhibited 85.00% accuracy, 90.00% sensitivity, and 80.00% specificity^11^. In another study on aorta lumen segmentation, 260 type B aortic dissection patients were enrolled and a mean Dice coefficient exceeding 90% was recorded^12^.

The accuracy of diagnosis of acute thoracic aortic dissection is high when CT images and a CNN are used, reaching 85–90%. However, the accuracy is also high when physicians use CT images for diagnosis (the scores obtained are: 94.90% accuracy, 82.60% sensitivity, and 100.00% specificity)^13^. This is because 3-dimensional images of the aorta and pathognomonic CT findings of type A acute thoracic aortic dissection are greatly helpful in diagnosis.

Moreover, CT images can be obtained hours after an emergency department (ED) visit, as intact kidney function must be confirmed with a blood test. Given that the mortality rates of acute thoracic aortic dissection increase by 2% per hour^14,15^, this time interval might be critical. Even if physicians decide to perform CT without checking renal function, performing CT becomes a challenge for all patients presenting chest pain because of radiation hazard and cost.

Chest x-rays are currently used as a screening tool in the ED because of the low cost, a 150 to 1350-fold lower radiation hazard^16,17^, and easy accessibility to the early phase of patients’ presentation in the ED. However, the sensitivity for screening acute thoracic aortic dissection is 70% for chest x-rays, which is not high enough^4,5^.

To our knowledge, there has been no study regarding improving the accuracy of acute thoracic aortic dissection with chest x-rays using CNNs. In this study, efforts are made to do that, and this may further improve patient outcomes.

## Methods

### Study design

This is a multicentre retrospective study aimed at learning and detecting aortic dissection using CNN to analyse chest X-ray images obtained from three tertiary academic hospitals (Seoul and Gyeonggi-do, Republic of Korea) between October 2021 and March 2022. The study was reported in accordance with the Checklist for Artificial Intelligence in Medical Imaging (CLAIM)^18.^

### Data collection

Data collected between 2003 and 2020 at Seoul National University Bundang Hospital (Hospital A) and between 2005 and 2020 at Hanyang University Hospital (Hospital B) were used to construct the training dataset. Considering the versatility of the trained CNN model, data collected between 2018 and 2020 at Yonsei University (Hospital C) was used as testing data, on which the proposed model had not been trained.

Chest X-ray images were obtained from three tertiary academic hospitals. One trained researcher in each institution who did not participate as an author investigated each patient's age, sex, major symptoms, final diagnosis, chest CT readings, and surgical records based on the electronic health records. Based on this, a list was prepared comprising patients whose final diagnoses were consistent with acute thoracic aortic dissection and who concurrently underwent chest CT. In reference to this list, chest x-rays collected during the initial hospital visit of all patients were collected from picture archiving and communication systems. In addition, the type of thoracic aortic dissection was classified following the readings of radiologists. Acute thoracic aortic dissection was classified based on chest CT readings or surgical records as Stanford type A and B—the former involves any part of the aorta proximal to the origin of the left subclavian artery and the latter involves the aorta distal to the left subclavian artery.

Positive images, i.e., chest X-ray images of patients with acute thoracic aortic dissection, were obtained from patients diagnosed with acute thoracic aortic dissection in the emergency department. Diagnosis of acute thoracic aortic dissection included only those confirmed by contrast-enhanced CT or surgical diagnosis. Chest X-ray images of patients who visited the emergency departments after surgery or endovascular treatment at other hospitals were excluded. Negative images were obtained from patients who visited the emergency department for chest pain but did not receive a specific diagnosis, such as aortic dissection, aortic aneurysm, heart failure, pneumothorax, or ischemic heart disease. These diagnoses were excluded by the emergency physician based on the patient's symptoms, laboratory studies, chest x-rays, and, if necessary, CT reports. For example, if a pneumothorax was suspected in the absence of air in the pleural space on a chest X-ray, a CT scan was performed to confirm it. Finally, chest X-rays of chest pain patients who were excluded from specific diagnoses depicting readings within the normal range were used as negative images. The obtained images exhibited a positive-to-negative ratio between 1:3 and 1:5. All chest X-ray images were extracted in Digital Imaging and Communications in Medicine (DICOM) format and converted to JPEG format via image pre-processing.

### Data pre-processing and augmentation

**Figure 1:**
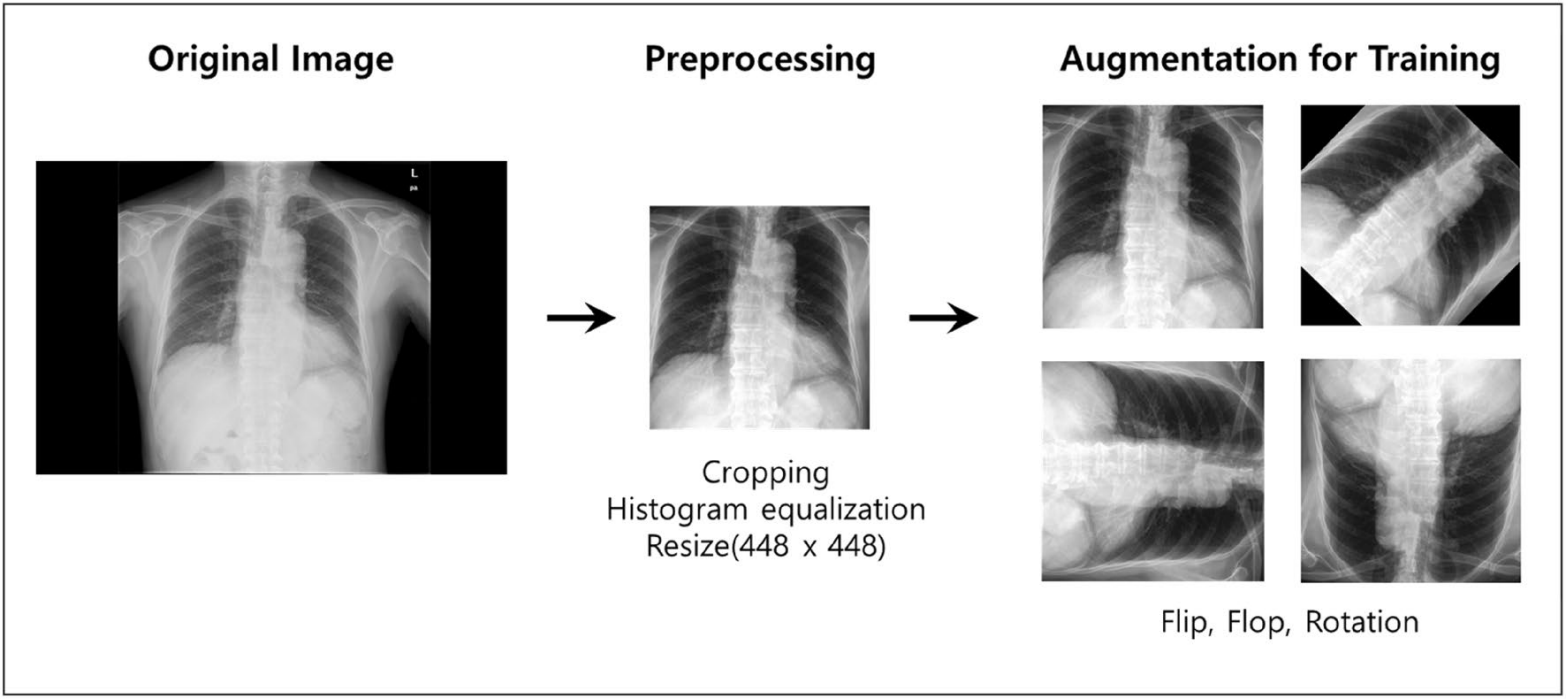
Image pre-processing.

Personal information, such as name, gender, and age, was removed from all chest X-ray images and only the images were used. Since chest X-ray images were collected from three different hospitals over an extended period of 18 years, a pre-processing step was necessary to ensure consistency (Fig. 1). First, unnecessary black margins were removed, and images were uniformly resized to a 448 × 448 pixel resolution. Next, the training images were augmented via random transformations, including flip, flop, and rotation—this transformation was needed to improve the performance of the model as the collected data included some images that had been flipped, flopped, and rotated. Further, a small amount of data was collected—thus, augmentation was needed to increase the size of the data and to secure robustness against geometric changes, such as scale, translational, and rotational transformation. Finally, histogram equalization, given by the following expression, was performed to reduce the deviation of the contrast:

$$T\left( {r_{k} } \right) = \frac{L}{n}\mathop \sum \limits_{j}^{k} n_{j}$$

$r_{k}$: Brightness of input image pixels, $n_{j}$: Number of $r_{k}$, L : Value of maximum brightness, n : Total number of pixels.

Histogram equalization yielded improved images by distributing the image brightness values such that uniform brightness values in the range between 0 and 255 were used.

### Diagnosis of acute thoracic aortic dissection via image classification

**Figure 2:**
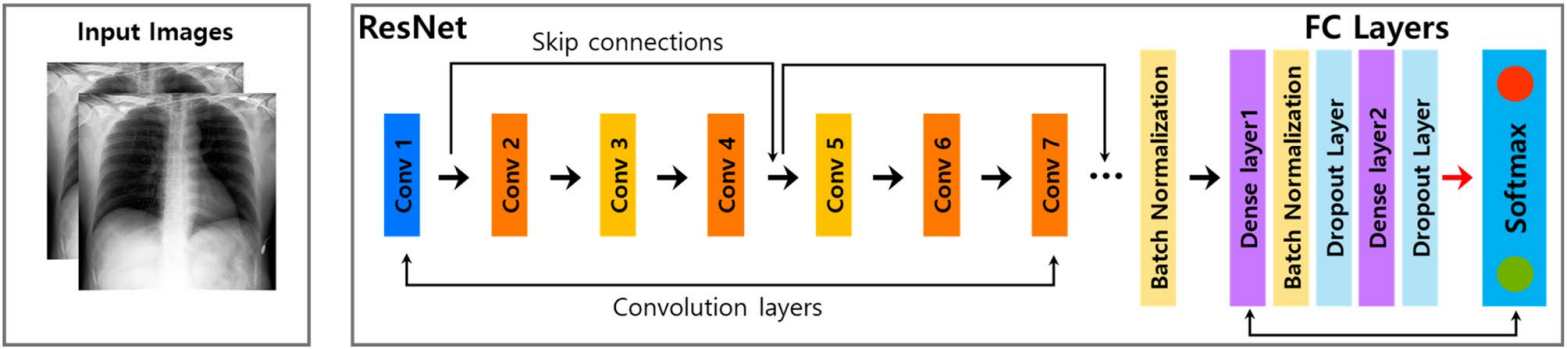
Model architecture of ResNet 18.

The proposed image classification architecture was based on residual neural network (ResNet) 18. ResNet is one of the most popular deep learning models in image classification, which successfully resolves the vanishing gradient problem, which is common during the training of traditional convolutional neural networks (CNNs) using residual mapping^19^. To construct the residual mapping, skip connections between layers were used to implement the network over multiple layers. The overall network architecture is illustrated in Fig. 2^20^.

**Figure 3:**
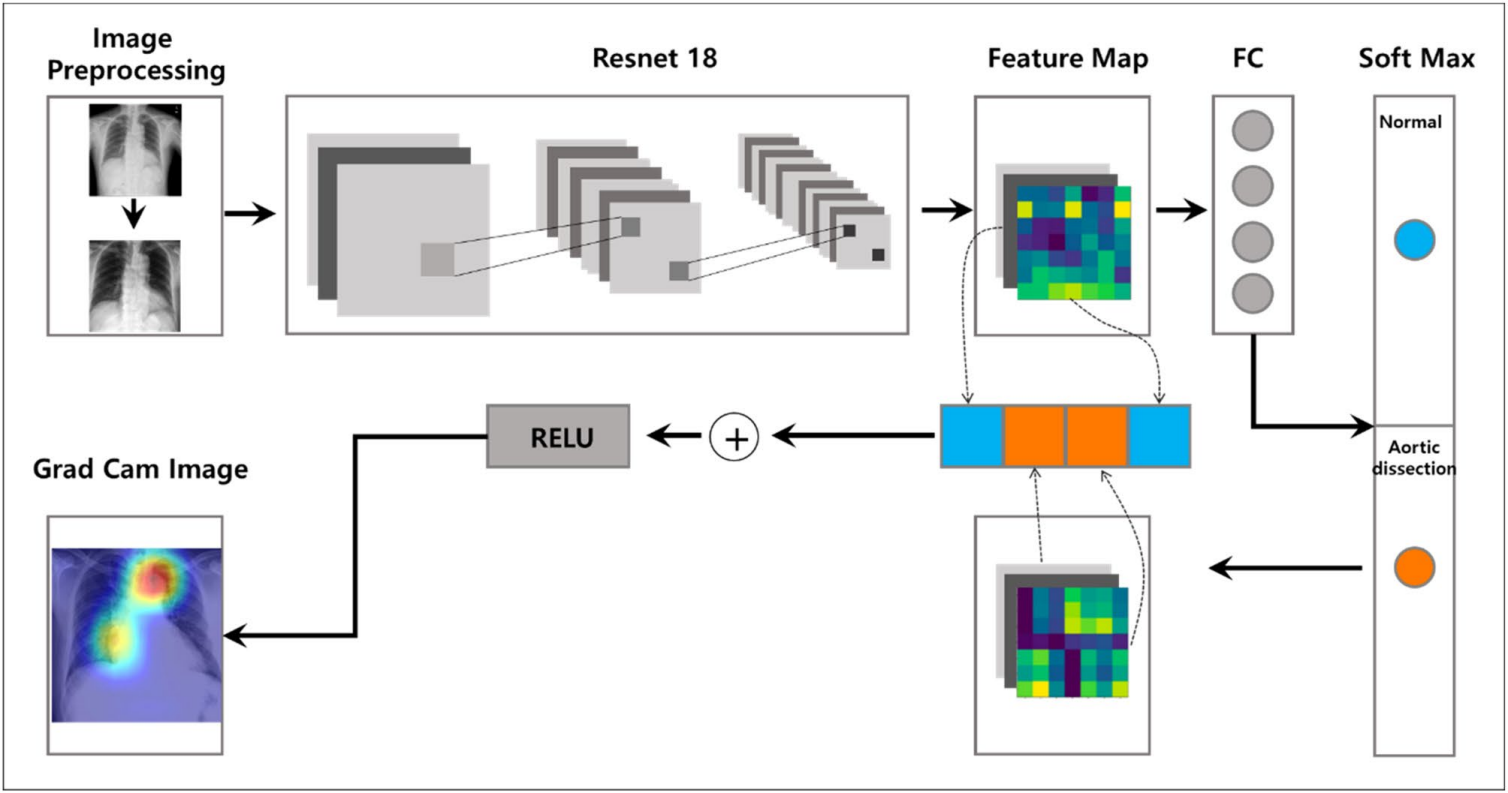
Overview of main architecture used for the diagnosis of aortic dissection.

The Resnet 18 methodology adopted for the detection and classification of aortic dissection is depicted in Fig. 3. The primary objective was to classify chest X-ray images into one of two categories—normal and aortic dissection. Two main stages were involved in the model—the pre-processing stage (which further included data augmentation and normalization) and the classification stage (which involved the use of Resnet18 on pre-trained models and prediction). The images were rescaled to 448 × 448 pixel resolution. Moreover, the images were augmented via: (1) rotation (2) horizontal flipping and vertical flipping.

During the training process of the Resnet18 model, performance degradation induced by data imbalance was mitigated by using a weighted random sampling method and a weighted cross-entropy loss function. In addition, an ensemble voting system was implemented to prevent overfitting.

The Gradient-weighted Class Activation Mapping (Grad-CAM) technique was employed to determine the aortic dissection detection transparency. This technique highlights the regions of the input image where the model pays greater attention during the classification process, implying that the feature maps generated in the final convolution layer contain the spatial information that aids the capture of the visual pattern. This visual pattern contributes to distinction between the assigned classes. The Grad-CAM technique was applied by utilizing the layers and extracted features of the trained model.

### Network performance and validation

The X-ray images collected from hospitals A and B were combined and used to construct a training dataset to train and validate the network, and the images from hospital C were used to construct the testing dataset to evaluate the performance of the network. K-fold (*k* = 5) cross-validation was performed on the final network architecture using the training dataset to achieve satisfactory performance while avoiding overfitting. During fivefold cross-validation, the training dataset was randomly divided into five roughly equal-sized subsets—four of them were used to train the network and the remaining one was used to estimate its performance.

**Figure 4:**
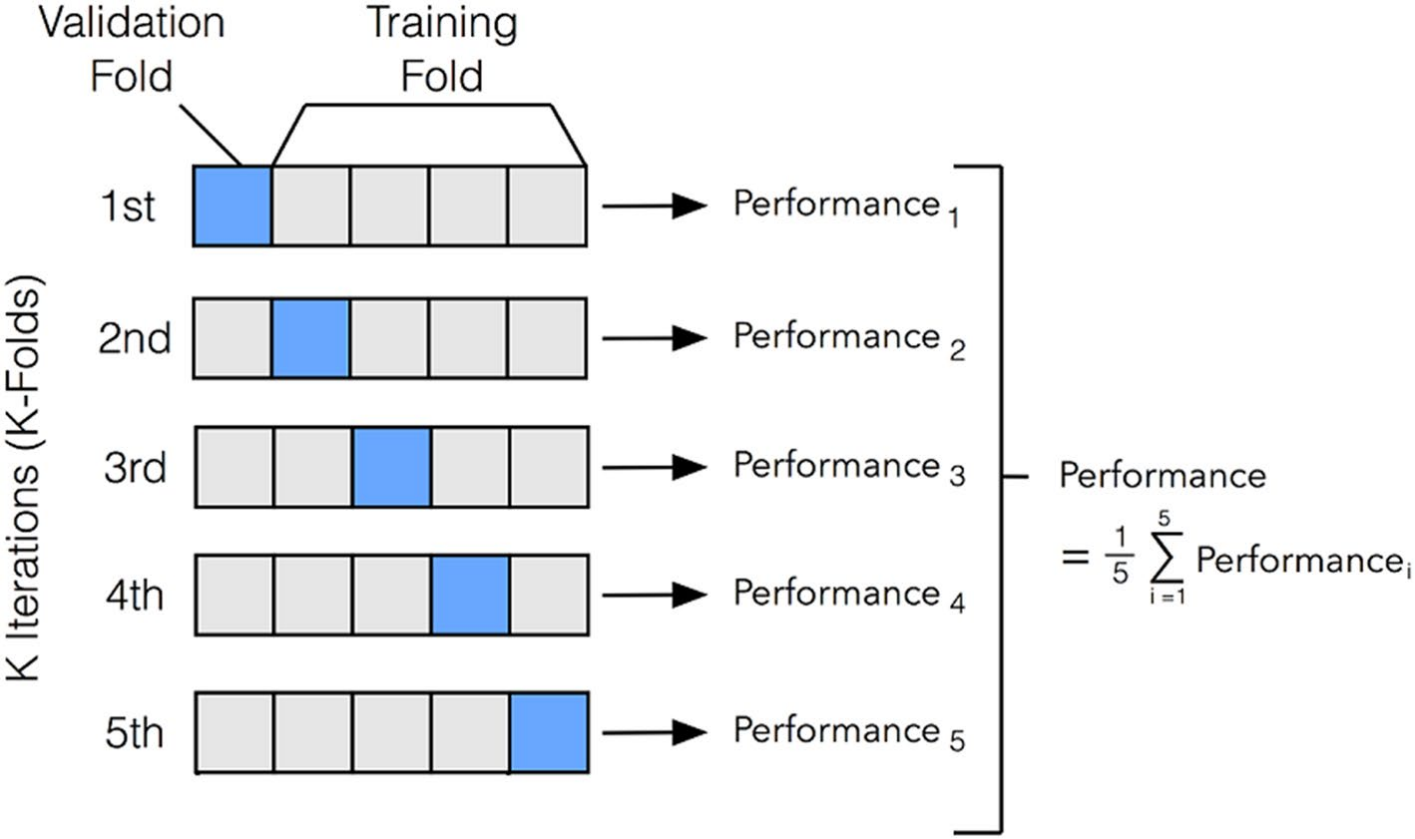
Ensemble voting system using fivefold cross-validation.

An ensemble voting system was used to further improve the robustness and reduce the risk of overfitting. In the proposed model, five models resulting from fivefold cross-validation were ensembled (Fig. 4). After using a soft voting classifier to assign probabilities to target variables, the training data were first shuffled and then divided into five groups, and passed to a fivefold training model. Individual predictions were obtained from each model using a voting aggregator and the soft voting technique. Finally, the majority vote was calculated, yielding the final prediction^21,22^.

### Data imbalance

In the collected training dataset, 20% of the images were positive images and the remaining 80% were negative images. To prevent performance degradation caused by data imbalance, a weighted random sampling method and a weighted cross-entropy loss function were used. Random sampling is a part of the sampling technique, in which each sample is assigned an equal probability of every mini-batch. In the mini-batch (size = 16) for learning, the ratio of the positive and negative images was set to 1:1. The weighted Cross-Entropy loss function was used to solve the negative effect of overfitting on the training dataset on the accuracy of the deep learning model due to a decrease in the imbalance of the convergence speed of the loss function^23^. The following standard weighted binary cross-entropy loss function was used:

$$J_{wbce} = - \frac{1}{M}\mathop \sum \limits_{m = 1}^{M} \left[ {w \times y_{m} \times \log \log \left( {h_{\theta } \left( {x_{m} } \right)} \right) + \left( {1 - y_{m} } \right) \times \log \log 1 - h_{\theta } \left( {x_{m} } \right) } \right)]$$

where $x_{m}$ and $y_{m}$ denote input and target labels during training, and $h_{\theta }$ denotes a model with neural network weights, $\theta$. Here, $w$ denotes a weight, which is taken to be 0.7 in the following experiment based on experience.

### Visual verification via grad-CAM

For visual verification of the diagnostic results of the proposed deep learning network, Grad-CAM was implemented on the final convolutional layer. Grad-CAM uses the gradients of any target classes passing through the final convolutional layer of the CNN to generate a highlighted localization map depicting the essential regions of the image for the prediction of acute thoracic aortic dissection. The class-discriminative localization map is given by:

$$L_{Grad - CAM}^{c} = ReLU\left( {\mathop \sum \limits_{k} \alpha_{k}^{c} A^{k} } \right)$$

where $A^{k}$ denotes feature map of the $k^{th}$ channel, and $\alpha_{k}^{c}$ represents partial linearization of the deep network, which is the primary function of the feature map.

### Primary outcomes and performance evaluation

The primary outcome of this study was the detection of acute thoracic aortic dissection based on chest X-ray scanning using a CNN model. To validate the performance of five models trained via fivefold cross-validation, accuracy, sensitivity, specificity, positive predictive value (PPV), negative predictive value (NPV), and F-1 score were calculated. In addition, the performance of the ensemble model, which yields the final prediction via soft voting of the five models created during the cross-validation process, on the testing dataset was estimated.

To evaluate the performance of the model, we calculated its precision, recall, F1-score, and accuracy. The normal case and acute thoracic aortic dissection were considered as negative and positive cases, respectively. True positives (TP), true negatives (TN), false positives (FP), and false negatives (FN) were estimate based on the confusion matrices. These were calculated using the following parameters and equations:

#### True positive (TP)

An image with acute thoracic aortic dissection is classified in the acute thoracic aortic dissection category.

#### True negative (TN)

A normal image is classified in the normal category.

#### False positive (FP)

A normal image is incorrectly classified in the aortic dissection category.

#### False negative (FN)

An image with acute thoracic aortic dissection is incorrectly classified in the normal category.

**Precision** denotes the fraction of correct positive detection of acute thoracic aortic dissection.

**Recall** represents the quality of all the positives, which depends on the percentage of total relevant cases correctly classified by the model.

**F1-score** denotes the harmonic mean of precision and recall.

$$\begin{aligned} Precision & = \frac{{TP}}{{TP + FP}} \\ Recall & = \frac{{TP}}{{FN + TP}} \\ Accuracy & = \frac{{TP + TN}}{{FP + TP + TN + FN}} \\ F1 - score & = 2 \times \frac{{Precision \times Recall}}{{Precision + Recall}} \\ \end{aligned}$$

### Statistical analysis

All data processing and statistical analyses were performed using the Pytorch (ver.1.6.0, https://pytorch.org) environment for Resnet construction, training, and evaluation. Kolmogorov–Smirnov tests were performed to demonstrate the normal distribution of all datasets. We generated descriptive statistics and presented them as frequency and percentage for categorical data and as either median and interquartile range (IQR) (non-normal distribution) or mean and standard deviation (SD) (normal distribution) or 95% confidence interval (95% CI) for continuous data. The AUC of the receiver operating characteristic (ROC) was used to measure the performance of the deep learning model. Two-tailed p < 0.05 was considered to be significantly different.

### Experimental environment

Weighted binary cross-entropy was adopted as the loss function to fit the binary classifier (weight for type A = 3:7, type B = 1:9), and Adam was used as the optimiser function (learning rate = 0.0001). Training and testing were performed using a GeForce RTX 2080 Ti GPU (NVIDIA, Santa Clara, CA, USA). The network weights were initialised based on a pre-trained model on Resnet18, and the network was trained end-to-end using stochastic gradient descent (SGD). We trained the model in batches of 16, with an initial learning rate of 0.0001, which was decreased by 0.5 gamma every 10 epochs.

### Ethics approval and consent to participate

This study was approved by the Institutional Review Board (IRB) of Seoul National University Bundang Hospital (B-2002/597–102), IRB of Hanyang University Hospital (2021–01-005), and IRB of Yonsei University Hospital (4–2022-0770). All methods and procedures were carried out in accordance with the Declaration of Helsinki.

## Results

**Figure 5:**
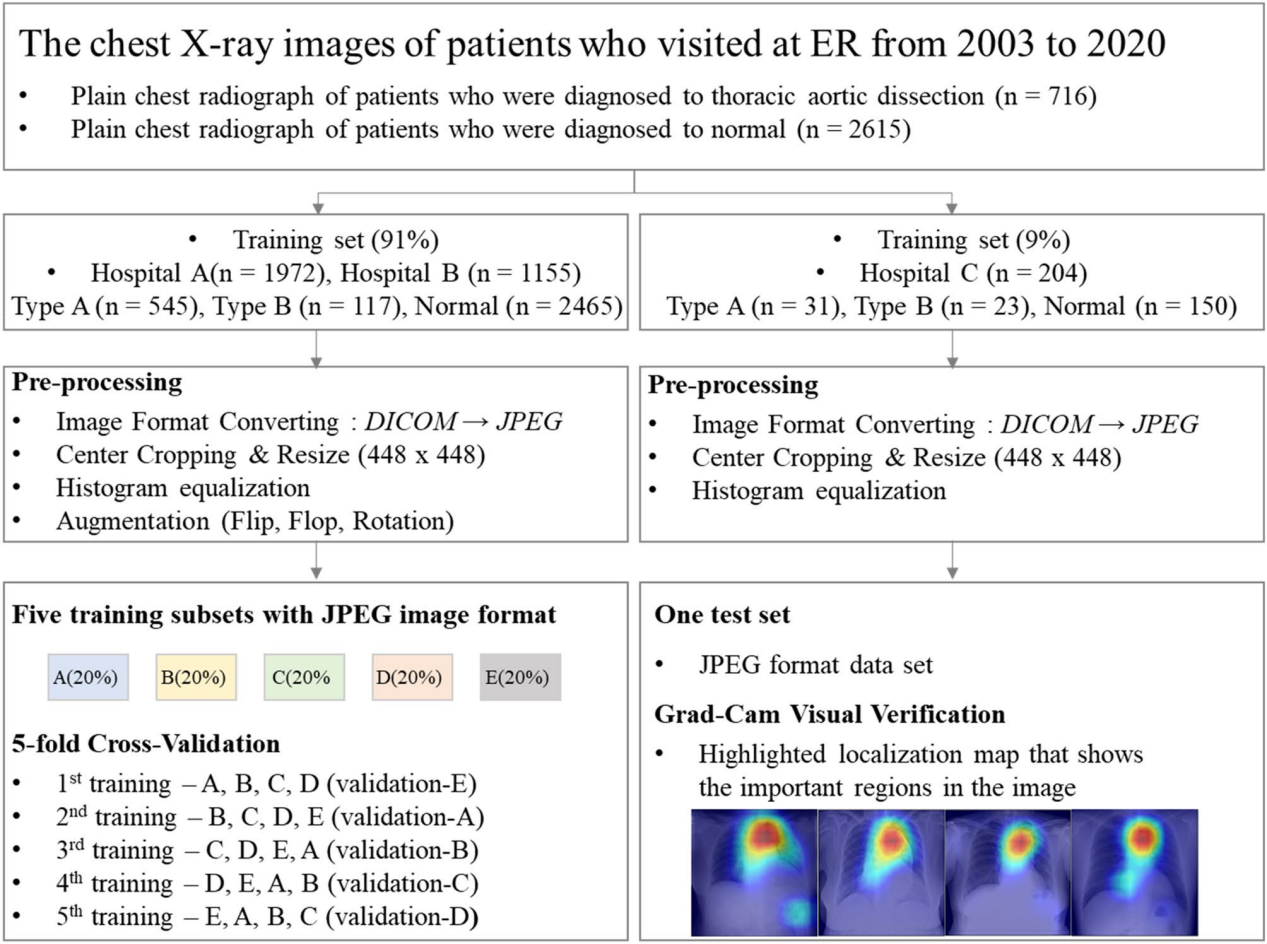
Flow chart of data collection and analysis during acute thoracic aortic dissection detection based on deep learning algorithms.

In aggregate, 3,331 images, containing 716 positive images and 2615 negative images, were collected from 3,331 patients. Overall, 1,972 images consisting of 507 positive images (gender: 62.7% male; age [SD]: 61 [15] years) from hospital A, 1,155 images consisting of 155 positive images (gender: 56.1% male; age [SD]: 63 [13] years), and 204 images consisting of 54 positive images (gender: 55.6% male; age [SD]: 61 [17] years) were analysed (Table 1). All patients with negative images visited the emergency department with chest pain and no specific diagnosis. 422 (83.2%), 123 (79.4%) and 31 (57.4%) patients were diagnosed with Stanford type A aortic dissection at hospitals A, B, and C, respectively. The datasets of hospitals A and B were separated into training data (80%) and internal validation data (20%) to verify the performance of the proposed method. The dataset of hospital C was used for testing (Fig. 5).

**Table 1:** Baseline characteristics of participants who provided images for the data sets.

	Aortic dissection			
	Type A + B	Type A	Type B	Control
Hospital A (*n* = 1972)	*n* = 507	*n* = 422	*n* = 85	*n* = 1465
Age, year, mean [SD]	61 [15]	61 [15]	63 [14]	59 [18]
Sex, male, *n* (%)	318 (62.7)	269 (63.7)	49 (57.6)	691 (47.2)
Hospital B (*n* = 1155)	*n* = 155	*n* = 123	*n* = 32	*n* = 1000
Age, year, mean [SD]	63 [13]	63 [13]	65 [13]	44 [11]
Sex, male, n (%)	87 (56.1)	66 (53.7)	21 (65.6)	526 (52.6)
Hospital C (*n* = 204)	*n* = 54	*n* = 31	*n* = 23	*n* = 150
Age, year, mean [SD]	61 [17]	63 [13]	65 [13]	39 [14]
Sex, male, *n* (%)	30 (55.6)	14 (45.2)	16 (69.6)	64 (42.7)
All (*n* = 3331)	*n* = 716	*n* = 576	*n* = 140	*n* = 2615
Age, year, mean [SD]	62 [15]	61 [15]	63 [14]	52 [17]
Sex, male, *n* (%)	435 (60.8)	349 (60.6)	86 (61.4)	1281 (49.0)

Continuous variables are presented by mean [standard deviation] and categorical variables are presented by N (%); *SD* standard deviation.

## Performance of the deep learning model

**Figure 6:**
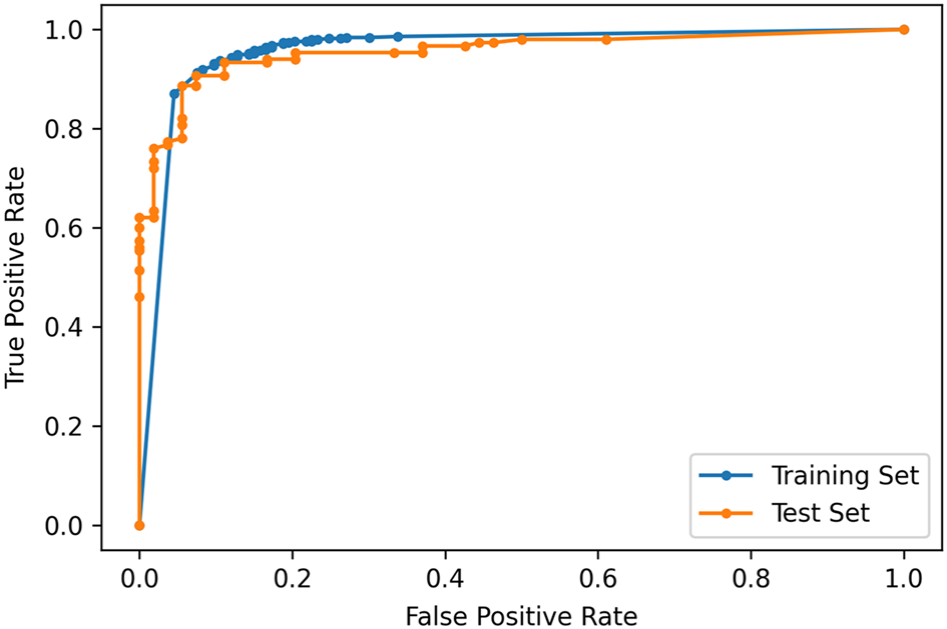
The ROC for the trained classification model. The AUC was 0.955.

The diagnostic performance matrix and outcomes are presented in Tables 2 and 3, respectively. The average accuracy on the validation set was 90.20%. The testing data set was obtained from data collected at hospital C, and the deep learning model was not trained on this data set. To evaluate the performance of the final deep learning model, the ROC curve was drawn and its AUC was calculated to be 0.955 on the testing data set (Fig. 6). The diagnostic accuracy of the deep learning model was 90.20%, with 75.00% precision, 94.44% recall, and 83.61% F1-score, on acute thoracic aortic dissection images.

**Table 2:** Diagnostic performance matrix.

	Internal validation					
	Onefold	Actual positive	Actual negative	Twofold	Actual positive	Actual negative
0.5 of Cut-off Value	Predicted positive	113	23	Predicted positive	112	31
	Predicted negative	20 (Type A 19/117, 16.3%; Type B 1/16, 6.2%)	470	Predicted negative	21 (Type A 17/114, 14.9%; Type B 4/19, 11.1%)	462

	Internal validation					
	Threefold	Actual positive	Actual negative	Fourfold	Actual positive	Actual negative
0.5 of Cut-off Value	Predicted positive	108	34	Predicted positive	119	38
	Predicted negative	24(Type A 21/105, 20.0%; Type B 3/27, 11.1%)	459	Predicted negative	13 (Type A 10/111, 9.1%; Type B 3/21, 14.3%)	455

	Internal validation			Test		
	Fivefold	Actual positive	Actual negative		Actual positive	Actual negative
0.5 of Cut-off Value	Predicted positive	117	38	Predicted positive	51	17
	Predicted negative	15 (Type A 14/109, 12.8%; Type B 1/23, 4.3%)	455	Predicted negative	3 (Type A 0/31, 0.0%; Type B 3/23, 13.1%)	133

Fivefold validation was performed with the dataset from hospital A and B. A hard voting method was used with five models from fivefold cross-validation to obtain the final classification result. Type A, type A aortic dissection; Type B, type B aortic dissection.

**Table 3:** Diagnostic performance matrix on the internal validation using fivefold validation and Test data set.

Training	Accuracy (%)	Precision (%)	Recall (%)	F1-score (%)	Test	Accuracy (%)	Precision (%)	Recall (%)	F1-score (%)
Onefold	93.13	83.09	84.96	84.01	Onefold	81.86	60.71	92.59	72.99
Twofold	91.69	78.32	84.21	81.16	Twofold	87.25	71.88	85.19	77.97
Threefold	90.72	76.06	81.82	78.83	Threefold	87.75	70.42	92.59	80.00
Fourfold	91.84	75.80	90.15	82.35	Fourfold	89.22	78.57	81.48	80.00
Fivefold	91.52	75.48	88.64	81.53	Fivefold	87.25	71.21	87.04	78.33
Average	91.78	77.75	85.96	81.57	Soft voting result	90.20	75.00	94.44	83.61
					Type A			100.00	
					Type B			86.96	

Fivefold validation was performed with the dataset from hospital A and B. A hard voting method was used with five models from fivefold cross-validation to obtain the final classification result.

## Regions of interest for aortic dissection

**Figure 7:**
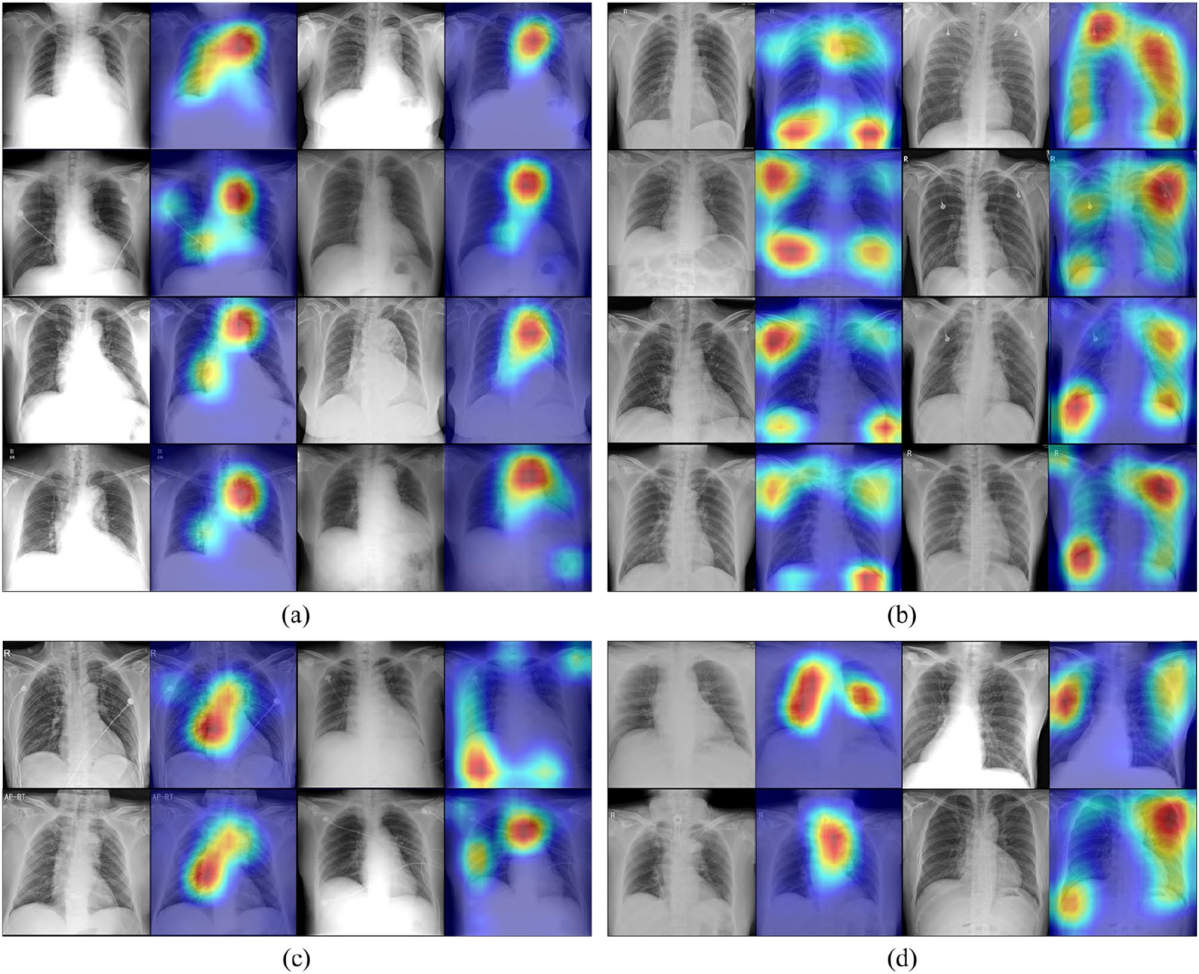
The regions of interest for aortic dissection diagnosis were visualised as heat maps based on Grad-CAM following the confusion matrix categories: (**a**) true positive, (**b**) true negative, (**c**) false positive, and (**d**) false negative.

Figure 7. depicts the classification results obtained using the deep learning network. The model trained using the results of the true positive category emphasised the aortic region. Conversely, the highlighted parts were scattered in the true negative category. In the false positive and false negative categories, cases focusing on the aorta and scattered regions were mixed.

## Discussion

Several studies have investigated the detection accuracy of aortic dissection by applying CNN on CT images. Since contrast chest CT uses a contrast material to enhance blood vessels, it is a modality of choice for the diagnosis of acute thoracic aortic dissection. It aids physicians to distinguish between an enhanced dissected aorta and a normal one. However, the detection of dissected aorta based on chest x-rays is more challenging due to the lack of enhancement of blood vessels, unlike in CT images. Therefore, in this study, we improved the diagnostic accuracy of aortic dissection based chest x-rays using a CNN. To the best of our knowledge, this is the first attempt to improve the diagnostic accuracy of aortic dissection by learning chest X-rays using a CNN. As chest X-ray is the most basic examination modality for patients visiting the emergency department with chest pain and a common screening test for aortic dissection, the proposed model is expected to facilitate clinical screening for aortic dissection.

To determine the model with the best performance, ResNet (18 and 34), DenseNet, and EfficientNet (b0 and b1) were used. Table 1 presents the number of parameters in each network and the accuracy on the test data set. DenseNet and EfficientNet (b0, b1) exhibited poorer performance in representative cases despite a slight increase in parameters compared to the original ResNet (18 and 34). ResNet 18 required fewer parameters than ResNet 34 and exhibited superior performance than the other models. Therefore, we used ResNet18 as our final model (Supplementary table 1).

In chest X-ray scanning images, the aorta is distinguished based on the contrast between the air-filled lungs and the fluid-filled aorta. The ascending aorta can be identified outside the upper-right cardiac silhouette on a normal chest X-ray image. Moreover, the aortic arch is typically small and distinct in the upper left mediastinum, and the descending aorta can be distinguished as a clean, crisp stripe to the left of the vertebral column on a normal chest X-ray image^24^.

In chest x-ray images, aortic dissection is characterised by certain features, including mediastinal widening, expansion of aortic diameter, presence of double density due to enlargement of the false lumen, irregular contour due to edema and haemorrhage in the tissues, blurred aortic knob, displacement of intimal calcium, discrepancy in diameters of ascending and descending aorta, displacement of trachea/left main bronchus/oesophagus, and pleural effusion^25^. Moreover, abnormal findings on the lung field are possible, which are indicative of impending aorta rupture, such as pneumonitis caused by transmural aortic bleeding, nonspecific inflammatory reaction, secondary bronchopneumonia, regional compression atelectasis, and para-aortic hematoma^26^.

Although X-ray scanning is a useful screening tool based on the aforementioned guidelines, its diagnostic accuracy is not satisfactory considering the fatality of aortic dissection. According to a meta-analysis, the sensitivity of chest X-ray scanning was 64% when evaluated based on wide mediastinum and 71% based on a combination with abnormal aortic contour^4^. Even though a sensitivity of 90% was achieved when all nonspecific abnormal findings, such as pleural effusion, were combined, the development of a more accurate method is required since it is difficult to assume that all patients with nonspecific abnormal findings on X-ray scanning suffer from aortic dissection.

Christoph et al. used transthoracic echocardiography (TTE), transoesophageal echocardiography (TEE), CT, and magnetic resonance image (MRI) to diagnose aortic dissection, achieving sensitivities of 59.3%, 97.7%, 93.8%, and 98.3%, respectively; accuracies of 69.8%, 90.0%, 91.1%, and 98.0%, respectively; and precisions of 81.4%, 87.7%, 91.8%, and 98.3%, respectively^27^. In the present study, chest X-ray images were analysed using a CNN to detect acute thoracic aortic dissection, yielding a sensitivity of 94.44% and an accuracy of 90.20%—thus the performance was comparable to those of TEE and CT, and better than that of TTE in terms of accuracy. Although the precision achieved was low, the results were deemed to be relevant considering the ease of obtaining chest X-ray images and using them as a screening test.

The results were also notable compared to manual chest x-ray readings of radiologists. William et al. reported a sensitivity of 86%, an accuracy of 60%, and a precision of 53% during the diagnosis of aortic dissection based on abnormal chest x-ray findings, which is lower than the result obtained using the proposed CNN^28^.

The high aortic dissection detection accuracy of the proposed CNN can be explained based on the heat maps. Since the area highlighted in the heat map of the true positive category surrounded the aortic knob, mediastinal widening, aortic diameter expansion, and double contour of the aorta, which are directly related to aortas, were focussed on by the CNN. Additionally, the aortic diameter tends to taper from the origin to the downstream region gradually^29^. If the diameter of ascending aorta or descending aorta is larger than the diameter of the origin, the possibility of aortic dissection must be considered even if the range is normal. There may be a possibility that these points were reflected in the CNN, which is why the achieved accuracy was higher than that of a radiologist.

Considering the aforementioned comparisons with other diagnostic tools and radiologists, the proposed deep learning algorithm for acute thoracic aortic dissection based on chest X-ray scanning can be considered to be a reliable, quick method to detect acute thoracic aortic dissection. Its application as a screening test on patients visiting the emergency department is expected to enable the detection of cases that might otherwise have been missed. Based on the preliminary result, immediate confirmatory tests may be performed.

The accurate identification of aortic contour based on medical images and detection of aortic dissection accordingly are challenging. In a previous report, aortic contours were automatically distinguished from non-contrast CT images^30^, and Hata et al. classified aortic dissection using ML in non-contrast CTs. They achieved an accuracy of 90.0% and a sensitivity of 91.8%^10^. Even though the identification of the aorta by applying ML algorithm on chest X-ray scanning was not performed in this study, our results are still compelling because the reported accuracy and sensitivity are as high as those of CT studies, including the accuracy of identification of the aorta.

Regarding aorta identification based on chest X-ray scanning, the attention mechanism, which is known to function well in image classification tasks by increasing the representation power, was utilised to increase the accuracy of the diagnostic network. As depicted in Fig. 3, the trained model emphasises the thoracic aortic region in the true positive category, while the highlighted parts are scattered in the true negative category. This indicates that the proposed deep learning model concentrates on the thoracic region during the detection of acute thoracic aortic dissection without distinguishing aortic contours in chest X-ray images. Further, in the false positive and false negative categories, a combination of cases of aortic concentration and vice versa were observed, which corroborates the emphasis on the aortic region without thoracic aortic classification. Given that abnormal X-ray findings in acute thoracic aortic dissection are not limited to the aorta, this result seems reasonable. However, further research is required to classify acute thoracic aortic dissection after segmenting the aortic contour.

## Limitations

This study suffers from certain limitations. Firstly, the age and sex of the patients were not completely matched to the presence of aortic dissection because aortic dissection is more prevalent corresponding to a certain age and sex. Secondly, radiographic images of assorted sizes taken in different environments were pre-processed and used as input images. Even though they were cropped around the aorta, except for the outer edges and margins of the image, the trained model could not identify the aorta properly in some images and sometimes misclassified it. Therefore, better results can be expected if classification is performed following aortic segmentation. Third, although equal proportions of the training dataset were allocated for acute thoracic aortic dissection and normal imaging, an imbalanced test dataset may reduce the reliability of the test results. Fourth, in this study, binary classification of normal images and acute thoracic aortic dissection images was performed without classifying the type of acute thoracic aortic dissection, and performance was not evaluated by including images of other aortic syndromes and other specific conditions causing chest pain. Finally, although the performance of the aortic dissection classification model was good, our observations are not sufficient to conclude that chest x-rays can be used to replace CT scans completely in patients with suspected aortic dissection.

## Conclusions

The detection accuracy of acute thoracic aortic dissection using Resnet 18 was 90.20%. This model is expected to facilitate the screening of patients with suspected acute thoracic aortic dissection among patients who visit the emergency department with chest pain. Given the high severity and acuity of thoracic aortic dissection, early suspicion based on chest X-rays and CNN could accelerate the diagnosis and improve the prognosis. In future works, the accuracy should be improved based on segmentation, and research directly applicable to clinical practice should be prioritised.

## Supplementary Information

Supplementary Information.

## Data Availability

The data presented in this study are available on request from the corresponding author.
