# Author Correction: Detection of acute thoracic aortic dissection based on plain chest radiography and a residual neural network (Resnet)

**DOI:** 10.1038/s41598-023-29407-0

**Published:** 2023-02-09

**Authors:** Dong Keon Lee, Jin Hyuk Kim, Jaehoon Oh, Tae Hyun Kim, Myeong Seong Yoon, Dong Jin Im, Jae Ho Chung, Hayoung Byun

**Affiliations:** 1 Department of Emergency Medicine, Seoul National University Bundang Hospital, Seongnam, Republic of Korea; 2 Department of Emergency Medicine, Seoul National University College of Medicine, Seoul, Republic of Korea; 3 Department of Computer Science, Hanyang University, 222 Wangsimni‑ro, Seongdong‑gu, Seoul, 04763 Republic of Korea; 4 Machine Learning Research Center for Medical Data, Hanyang University, Seoul, Republic of Korea; 5 Department of Emergency Medicine, College of Medicine, Hanyang University, 222 Wangsimni‑ro, Seongdong‑gu, Seoul, 04763 Republic of Korea; 6 Department of Radiology and Research Institute of Radiological Science, Severance Hospital, Yonsei University College of Medicine, Seoul, Republic of Korea; 7 Department of Otolaryngology-Head and Neck Surgery, College of Medicine, Hanyang University, Seoul, Republic of Korea; 8 Department of HY, College of Medicine, KIST Bio-Convergence, Hanyang University, Seoul, Republic of Korea

Correction to: *Scientific Reports* 10.1038/s41598-022-26486-3, published online 19 December 2022

The original version of this Article contained an error in the spelling of the author Jin Hyuk Kim which was incorrectly given as Kim Jin Hyuk.

Consequently, the equal contribution statement was incorrect.

“These authors contributed equally: Dong Keon Lee, Kim Jin Hyuk, Jaehoon Oh and Tae Hyun Kim.”

now reads:

“These authors contributed equally: Dong Keon Lee, Jin Hyuk Kim, Jaehoon Oh and Tae Hyun Kim.”

The original Article has been corrected.

