# Supplementary material for: Detection of acute thoracic aortic dissection based on plain chest radiography and a residual neural network (Resnet)
Source: Sci Rep. 2022 Dec 19;12:21884. doi: 10.1038/s41598-022-26486-3 (PMC9763432; doi:10.1038/s41598-022-26486-3)
Supplement: Supplementary file 1 — Supplementary Information. [file 41598_2022_26486_MOESM1_ESM.docx]

Supplementary table 1. Comparison of the diagnostic accuracy between the current network (ResNet18) and other available machine learning networks

|  | **Resnet 18** | **Resnet 34** | **Densenet** | **EfficientNet-b0** | **EfficientNet-b1** |
| --- | --- | --- | --- | --- | --- |
| **Accuracy (%)** | 90.20 | 87.33 | 85.93 | 88.21 | 86.89 |
| **Number of parameters** | 11.1M | 21.2M | 7.6M | 4.0M | 6.5M |
